# Clinical outcomes and mortality associated with weekend admission to psychiatric hospital

**DOI:** 10.1192/bjp.bp.115.180307

**Published:** 2016-07

**Authors:** Rashmi Patel, Edward Chesney, Alexis E. Cullen, Alex D. Tulloch, Matthew Broadbent, Robert Stewart, Philip McGuire

**Affiliations:** **Rashmi Patel**, BM BCh, **Edward Chesney**, BM BCh, Department of Psychosis Studies, Institute of Psychiatry, Psychology & Neuroscience, King's College London, London; **Alexis E. Cullen**, PhD, Department of Psychosis Studies and Department of Health Service and Population Research, Institute of Psychiatry, Psychology & Neuroscience, King's College London, London; **Alex D. Tulloch**, PhD, Department of Health Service and Population Research, Institute of Psychiatry, Psychology & Neuroscience, King's College London, London; **Matthew Broadbent**, BSc, Biomedical Research Centre Nucleus, South London and Maudsley NHS Foundation Trust, London; **Robert Stewart**, MD, Department of Psychological Medicine, Institute of Psychiatry, Psychology & Neuroscience, King's College London, London; **Philip McGuire**, PhD, Department of Psychosis Studies, Institute of Psychiatry, Psychology & Neuroscience, King's College London, London, UK

## Abstract

**Background**

Studies indicate that risk of mortality is higher for patients admitted to acute hospitals at the weekend. However, less is known about clinical outcomes among patients admitted to psychiatric hospitals.

**Aims**

To investigate whether weekend admission to a psychiatric hospital is associated with worse clinical outcomes.

**Method**

Data were obtained from 45 264 consecutive psychiatric hospital admissions. The association of weekend admission with in-patient mortality, duration of hospital admission and risk of readmission was investigated using multivariable regression analyses. Secondary analyses were performed to investigate the distribution of admissions, discharges, in-patient mortality, episodes of seclusion and violent incidents on different days of the week.

**Results**

There were 7303 weekend admissions (16.1%). Patients who were aged between 26 and 35 years, female or from a minority ethnic group were more likely to be admitted at the weekend. Patients admitted at the weekend were more likely to present via acute hospital services, other psychiatric hospitals and the criminal justice system than to be admitted directly from their own home. Weekend admission was associated with a shorter duration of admission (*B* coefficient −21.1 days, 95% CI −24.6 to −17.6, *P*<0.001) and an increased risk of readmission in the 12 months following index admission (incidence rate ratio 1.13, 95% CI 1.08 to 1.18, *P*<0.001), but in-patient mortality (odds ratio (OR) = 0.79, 95% CI 0.51 to 1.23, *P* = 0.30) was not greater than for weekday admission. Fewer episodes of seclusion occurred at the weekend but there was no significant variation in deaths during hospital admission or violent incidents on different days of the week.

**Conclusions**

Being admitted at the weekend was not associated with an increased risk of in-patient mortality. However, patients admitted at the weekend had shorter admissions and were more likely to be readmitted, suggesting that they may represent a different clinical population to those admitted during the week. This is an important consideration if mental healthcare services are to be implemented across a 7-day week.

Variation in clinical outcomes of patients who are admitted at the weekend has been reported in healthcare services around the world.^1–7^ Studies in hospitals providing acute medical, surgical and obstetric care suggest that patients admitted to hospital at the weekend are at increased risk of mortality.^3–6,8^ It is thought that this may be partly explained by varying availability of healthcare services on different days of the week, and by differences in the clinical characteristics of patients who are admitted at the weekend.^9^ In the UK, these findings have led to an aspiration to improve availability of healthcare at the weekends.^5^ However, to date, the literature in this field has involved studies in physical healthcare: the extent to which these findings apply to mental healthcare is unknown.

In the UK, around 10% of direct healthcare spending contributes towards mental health services.^10^ This include services for people requiring specialist mental healthcare in the community and in psychiatric hospitals.^10^ With the exception of home treatment teams, community services typically provide routine mental healthcare during the working week only.^11^ In-patient care in psychiatric hospitals is provided throughout the week but with reduced scheduled activity at the weekend. The extent to which variation in mental healthcare demand and service provision on different days of the week affects clinical outcomes is unclear. Using anonymised electronic health record data from a large provider of secondary mental healthcare, we sought to investigate whether patients admitted to a psychiatric hospital at the weekend had worse clinical outcomes, as indexed by an increased risk of mortality, longer duration of hospital admission or increased risk of readmission. We also investigated the distribution of adverse clinical outcomes including death, seclusion and violent incidents on different days of the week to determine whether these outcomes occurred more frequently at the weekend.

## Method

### Participants and source of clinical data

The study was conducted using anonymised electronic health record data from patients admitted to a psychiatric hospital in the South London and Maudsley NHS Foundation Trust (SLaM). SLaM is a large provider of mental healthcare covering a catchment population of around 1.2 million people in South London and provides specialist in-patient and community care for people with a wide range of mental disorders. The study included data from all hospital admissions of adults (aged 16 years and older) to a psychiatric hospital in SLaM between 1 April 2006 and 31 March 2015. Data from 45 264 hospital admissions were obtained and included in the present study.

The data were obtained from the SLaM Biomedical Research Centre (BRC) Case Register,^12^ a large data-set containing pseudonymised clinical data of over 250 000 patients.^13^ The BRC Case Register data include structured fields (for demographic, hospital admission and mortality data) and de-identified unstructured free-text fields from case notes and correspondence. Data for this study were obtained using the Clinical Record Interactive Search tool (CRIS), a database query and assembly tool that has supported a range of studies^14–17^ using clinical data from the SLaM BRC Case Register. The CRIS data resource received ethical approval as an anonymised data-set for secondary analyses from Oxfordshire REC C (Ref: 08/H0606/71+5).

### Exposure and outcome measures

The exposure (hereafter referred to as weekend admission) was defined as whether the beginning of a hospital admission occurred on a Saturday, Sunday or a UK bank holiday.^18^

The primary outcome measure was death during hospital admission. Secondary outcome measures were the duration of hospital admission and number of readmissions to hospital in the 12 months following discharge. These outcome measures were chosen as mortality, duration of admission and risk of readmission represent significant clinical outcomes for patients admitted to a psychiatric hospital and complete data on these outcomes were available from structured text fields for all 45 264 admissions (between 1 April 2006 and 31 March 2015) included in the study.

Further outcome data on episodes of seclusion and violent incidents were obtained from a subset of admissions to a psychiatric intensive care unit (PICU) in SLaM. PICUs are specialist in-patient wards that have higher staffing levels and provide intensive therapy to patients with serious mental illness who have a significant risk of harm to self or others and cannot be managed in a lower security environment.^19^ Patients who have acutely disturbed behaviour may be temporarily confined in a locked room under direct supervision (in a process known as seclusion) in order to help de-escalate the situation and reduce the risk of harm to self or others.^20^ Data were obtained from a separate study investigating the outcomes of seclusion in PICUs in SLaM^21^ by manually coding unstructured free text from in-patient progress notes and correspondence and comprised (a) episodes of seclusion occurring within PICU admissions ending between 1 April 2006 and 31 March 2013 and (b) violent incidents occurring within 1 week of a set of dates randomly sampled from PICU admissions starting between 1 April 2008 and 31 March 2013, excluding any days spent in seclusion. Free-text progress notes and correspondence were selected for inspection and coding as a possible day in seclusion if they contained the phrases “solitary confinement”, “supervised confinement” or “seclusion”, and were selected for inspection and coding as possible violent incidents if they contained any of the terms “Abus*”; “Aggress*”; “Agitat*”; “Arous*”; “Assault*”; “Attack*”; “Demand*”; “Hit*”; “Irritable”; “Manic”; “Punch*”; “Push*”; “Slap*”; “Refus*”; “Restrain*”; “Shout*”; “Threat*”; “Threw/Throw*”; and “Violen*”.

### Covariates

The following categorical variables were obtained as covariates for multivariable regression analyses: age, gender, ethnicity, mode of admission (whether admitted compulsorily under the UK Mental Health Act), source of admission and length of admission. All variables were obtained closest to the date of hospital admission. Ethnicity was recorded according to categories defined by the UK Office for National Statistics.^22^ Source of admission was recorded according to the following categories: home (including private residence, supported accommodation, residential and nursing homes); acute hospital (including emergency departments and any hospital services for patients receiving medical or surgical treatment); other psychiatric hospital; criminal justice system (including patients admitted from police custody or by a court order); other. Complete data on these covariates were available for all 45 264 admissions analysed in the study.

### Statistical analysis

Stata (version 12.0) was used to analyse the data. Univariate and multivariable binary logistic regression was used to investigate the association of age, gender, ethnicity, mode of admission and source of admission with weekend admission. The association of weekend admission was analysed with (a) death during admission using multivariable binary logistic regression, with (b) length of admission using multiple linear regression and with (c) number of readmissions using multivariable negative binomial regression. All multivariable analyses were adjusted for age, gender, ethnicity, mode of admission (whether admitted compulsorily under the UK Mental Health Act) and source of admission. The analyses of death during admission and number of readmissions were adjusted additionally with length of admission on the basis that an increased length of admission would necessarily increase time at risk for death during admission and is also associated with risk of future hospital admissions. Reference categories for covariates in multivariable analyses were defined as the category with the greatest prevalence within each group.

In order to investigate the distribution of outcomes across the 7-day week, descriptive statistics for number of in-patient deaths, admissions, discharges, seclusions and violent incidents were obtained for each day of the week (including bank holidays). A chi-squared goodness-of-fit test was used to identify whether there was any significant difference in the number of outcome events occurring on different days of the week with a null hypothesis that events are uniformly distributed across the week. For each analysis, Cohen's *w* (a measure of the minimum detectable effect size for chi-squared tests) was estimated at a significance level of 0.05 and power of 0.8 using the ‘pwr’ package in R (version 3.2.3). A Cohen's *w* of 0.1 represents a small effect size, 0.3 a medium effect size and 0.5 a large effect size.^23^

## Results

### Factors associated with weekend hospital admissions

Table 1 summarises the association of demographic and clinical factors with weekend admission. Patients aged over 65 years were least likely to be admitted at the weekend whereas patients aged 26–35 years were most likely. Female patients were more likely to be admitted at the weekend than male patients. Patients from an ethnic minority were more likely to be admitted at the weekend than White patients. Although there was no significant association of mode of admission with weekend admission on univariate analysis, after adjusting for other demographic and clinical factors compulsory admission was less likely at the weekend than during the week. Patients admitted at the weekend were substantially less likely to be admitted from home compared with other sources.

**Table 1:** Binary logistic regression analysis of factors associated with weekend psychiatric hospital admission (*n* = 45 264)

	Sample, *n*	Admitted on a weekend, %	Unadjusted OR (95% CI)	*P*	Adjusted OR (95% CI)^a^	*P*
Age, years						
16–25	4730	15.8	0.98 (0.89 to 1.07)	0.66	0.88 (0.80 to 0.97)	0.01
26–35	9448	19.1	1.23 (1.14 to 1.32)	<0.001	1.15 (1.07 to 1.24)	<0.001
36–45	11 218	16.1	Reference		Reference	
46–55	10 667	15.9	0.98 (0.92 to 1.06)	0.66	1.02 (0.95 to 1.10)	0.61
56–65	4548	16.0	0.99 (0.90 to 1.09)	0.87	1.02 (0.93 to 1.13)	0.65
>65	4653	11.2	0.65 (0.59 to 0.73)	<0.001	0.68 (0.61 to 0.75)	<0.001

Gender						
Female	20 816	16.5	1.05 (1.00 to 1.10)	0.06	1.06 (1.01 to 1.12)	0.02
Male	24 448	15.8	Reference		Reference	

Ethnicity						
White	25 959	15.1	Reference		Reference	
Asian	2124	15.8	1.05 (0.93 to 1.19)	0.44	1.00 (0.89 to 1.14)	0.95
Black	14 801	17.6	1.20 (1.31 to 1.26)	<0.001	1.13 (1.06 to 1.19)	<0.001
Other ethnic group	2380	18.4	1.26 (1.13 to 1.41)	<0.001	1.14 (1.02 to 1.27)	0.02

Mode of admission						
Admitted voluntarily	32935	16.2	Reference		Reference	
Admitted compulsorily	12 329	15.9	0.98 (0.92 to 1.03)	0.42	0.85 (0.80 to 0.91)	<0.001

Source of admission						
Home	18 262	8.1	Reference		Reference	
Acute hospital	14 668	25.1	3.79 (3.55 to 4.05)	<0.001	3.76 (3.52 to 4.01)	<0.001
Other psychiatric hospital	4112	21.5	3.10 (2.83 to 3.40)	<0.001	3.09 (2.82 to 3.39)	<0.001
Criminal justice system	1983	19.9	2.81 (2.49 to 3.18)	<0.001	2.88 (2.53 to 3.27)	<0.001
Other	6239	13.9	1.83 (1.67 to 2.00)	<0.001	1.75 (1.60 to 1.92)	<0.001

a. Adjusted for age, gender, ethnicity, mode of admission (whether admitted compulsorily under the UK Mental Health Act) and source of admission.

### Association of weekend admission with in-patient deaths, length of admission and risk of readmission

Table 2 summarises the multivariable regression analyses investigating the association of weekend admission with (a) risk of death during admission, (b) length of admission and (c) number of readmissions during the 12 months following discharge. Fewer in-patient deaths occurred among patients admitted at the weekend (3.1 per 1000 admissions) compared with patients admitted during the week (6.8 per 1000 admissions). Although there was a significantly reduced risk of death during admission for patients admitted at the weekend on univariate analysis (odds ratio (OR) = 0.46, 95% CI 0.30–0.71, *P*<0.001), this reduction was no longer significant after adjusting for demographic and clinical variables in multivariable analysis (OR = 0.79, 95% CI 0.51–1.23, *P* = 0.30). The length of hospital stay for patients admitted at the weekend (mean: 38.7 days, s.d. = 86.2, median: 16 days, interquartile range (IQR) = 4–40) was shorter than for patients admitted during the week (mean: 64.7, s.d. = 147.7, median 24 days, IQR = 8–63). This difference remained significant after adjusting for demographic and clinical factors in multiple linear regression analysis. Patients admitted at the weekend had an increased frequency of readmission in the 12 months following discharge (mean: 0.72 readmissions, s.d. = 1.24) compared with patients admitted during the week (mean: 0.59 readmissions, s.d. = 1.08). This difference remained significant after adjusting for demographic and clinical factors in multivariable negative binomial regression analysis.

**Table 2:** Association of weekend psychiatric hospital admission with risk of death during admission, length of admission and number of readmissions during the 12 months following discharge (*n* = 45 264)

	Death during admission^a^		Length of admission^b^		Number of readmissions^a^	
Model	OR (95% CI)	*P*	*B* coefficient, days (95% CI)	*P*	Incidence rate ratio (95% CI)	*P*
Univariate	0.46 (0.30 to 0.71)	<0.001	−26.0 (−29.5 to −22.5)	<0.001	1.22 (1.17 to 1.28)	<0.001

Multivariable	0.79 (0.51 to 1.23)	0.30	−21.1 (−24.6 to −17.6)	<0.001	1.13 (1.08 to 1.18)	<0.001

a. Multivariable analysis adjusted for age, gender, ethnicity, mode of admission (whether admitted compulsorily under the UK Mental Health Act), source of admission and length of admission.

b. Multivariable analysis adjusted for age, gender, ethnicity, mode of admission (whether admitted compulsorily under the UK Mental Health Act) and source of admission.

### Distribution of in-patient deaths, admissions, discharges, seclusions and violent incidents by day of week

There were 7303 weekend admissions out of a total of 45 264 admissions analysed in the study. Of these, 3794 occurred on a Saturday, 3020 on a Sunday and 489 on a UK bank holiday. Table 3 shows the distribution of in-patient deaths, admissions, discharges, seclusions and violent incidents across the week. In total, 281 deaths occurred during admission at a rate of 6.2 deaths per 1000 admissions. There was no statistically significant variation in deaths by day of week. The analysis of distribution of deaths by day of week was powered to detect a minimum effect size (Cohen's *w*) of 0.22. There were significantly fewer admissions on a Saturday and Sunday compared with other days of the week. There were also substantially fewer discharges on a Saturday and Sunday compared with other days of the week. There were significantly fewer episodes of seclusion on a Saturday and Sunday compared with other days in the week but no significant variation in violent incidents between different days of the week. The analysis of distribution of violent incidents by day of week was powered to detect a minimum effect size (Cohen's *w*) of 0.12.

**Table 3:** Distribution of in-patient deaths, admissions, discharges, seclusions and violent incidents by day of week

	*n*, %								MDE, Cohen's *w*	Goodness-of-fit, χ^2^(*P*)
	Monday	Tuesday	Wednesday	Thursday	Friday	Saturday	Sunday	Total		
In-patient deaths^a^	44 (15.7)	39 (13.9)	49 (17.4)	41 (14.6)	37 (13.2)	30 (10.7)	41 (14.6)	281 (100.0)	0.22	5.2 (0.52)

Admissions^a^	6821 (15.1)	7982 (17.6)	7595 (16.8)	7958 (17.6)	8094 (17.9)	3794 (8.4)	3020 (6.7)	45264 (100.0)	0.02	4266.7 (<0.001)

Discharges^a^	8993 (19.9)	9414 (20.8)	6432 (14.2)	9268 (20.5)	8671 (19.2)	1378 (3.0)	1108 (2.4)	45264 (100.0)	0.02	12741.0 (<0.001)

Seclusions^b^	256 (15.0)	272 (15.9)	262 (15.4)	247 (14.5)	247 (14.5)	197 (11.5)	225 (13.2)	1706 (100.0)	0.09	15.8 (0.02)

Violent incidents^c^	149 (14.9)	125 (12.5)	147 (14.7)	148 (14.8)	155 (15.5)	137 (13.7)	140 (14.0)	1001 (100.0)	0.12	4.1 (0.66)

MDE, minimum detectable effect.

a. Between 1 April 2006 and 31 March 2015 in any hospital ward.

b. Between 1 April 2006 and 31 March 2013 in a psychiatric intensive care unit.

c. Between 1 April 2008 and 31 March 2013 in a psychiatric intensive care unit.

## Discussion

This is the first study to investigate clinical outcomes associated with weekend admission to a psychiatric hospital and the distribution of adverse outcomes across the week. Our findings suggest that fewer admissions occur at the weekend than during the week. This is in keeping with findings from studies of admissions in acute hospitals providing medical and surgical care.^1,5^ We found substantially fewer discharges at the weekend compared with weekdays. These findings suggest that the majority of psychiatric hospital admissions and discharges occur during the week, when both community and in-patient services have higher staffing levels than at the weekend.

We found that patients who were young (aged 26–35 years), female and from an ethnic minority were more likely to be admitted at the weekend. This suggests that the sociodemographic characteristics of patients who are admitted at the weekend are different to those admitted during the week, consistent with data on weekend admissions to acute hospitals.^9^ After adjusting for demographic and clinical factors, compulsory admission was less common at the weekend than during the week. This could be the result of the process of enacting compulsory admission under the UK Mental Health Act, which requires the input of a large number of mental healthcare and allied professionals including two section 12 approved clinicians, an approved mental health professional and possibly also paramedics, the police and a locksmith (if it is deemed necessary to forcibly gain entry to a patient's private residence to conduct an assessment).^24^ The availability of staff required to perform a Mental Health Act assessment may be reduced at the weekend compared with during the week, resulting in a greater proportion of compulsory admissions during the working week.

The most significant association with weekend admission was that of source of admission. The majority of patients admitted from home (91.9%) were admitted during the week whereas a greater proportion of patients admitted from other sources were admitted at the weekend. This may reflect the fact that the typical referral pathway for a patient admitted to a psychiatric hospital from home involves presentation to a community service that is only available during the week. At the weekend, patients in the community can only be admitted to a psychiatric hospital via out-of-hours services, emergency departments and wards in acute hospitals, and from police custody.^11^ It is possible that the reduced availability of community services at the weekend may contribute towards an increase in admission via sources such as acute hospitals and the criminal justice system.

The overall rate of in-patient deaths was lower in our study (6.2 deaths per 1000 admissions) compared with a previous study investigating acute hospitals in England providing physical healthcare (46 deaths within 30 days per 1000 admissions^1^) where patients are more likely to be admitted with immediately life-threatening medical disorders. We did not find a statistically significant variation in the distribution of in-patient deaths on different days of the week and our analysis (which included 281 deaths) was powered to detect a small to medium minimum effect size. For this reason it is possible that our study lacked the power to detect a difference in distribution of deaths across the week, although we found that the fewest number of deaths occurred on a Saturday (30) and the greatest number occurred on a Wednesday (49). To determine if there is a genuine difference in psychiatric hospital in-patient mortality on different days of the week would require larger studies combining data from several mental healthcare providers.

We found that weekend admission was associated with a reduced likelihood of in-patient death in a univariate analysis but this reduction was no longer significant after adjusting for clinical factors in a multivariable analysis. This is in contrast to previous studies in acute hospitals that suggest an increase in mortality associated with weekend admission.^1,3–6,8^ This finding could be explained by the fact that in our study patients aged over 65 years, who may have medical comorbidities that increase their risk of mortality, were less likely to be admitted to a psychiatric hospital at the weekend.

We found that there were fewer seclusion episodes at the weekend compared with during the week, consistent with previous studies.^25–27^ One possible explanation for this finding is that there are fewer admissions at the weekend than during the week. Mental disorder symptom severity and aggressive behaviour are greatest within the first few days of the admission and improve with treatment,^28^ potentially resulting in a reduced need for the use of seclusion at the weekend compared with during the week. Indeed, a study examining factors associated with seclusion initiation indicate that the risk of seclusion is highest during the first 7 days of admission to PICU.^21^ Another contributing factor might be variation in aggressive behaviour and violent incidents on different days of the week that could be associated with an episode of seclusion. However, we found that there was no significant difference in the distribution of documented violent incidents on different days of the week and our analysis (which included 1001 violent incidents) was powered to detect a small minimum effect size. An alternative explanation for the reduced use of seclusion at the weekend might be that placing a patient in seclusion involves significant clinical resources to continually monitor the patient during the episode of seclusion. A reduction in the number of staff on in-patient wards at the weekend could therefore be associated with a reduced incidence of weekend seclusion. Further study investigating the association of violent incidents and staffing levels on wards with seclusion use would be necessary to test this hypothesis.

We investigated the association of weekend admission with length of admission and number of readmissions in the 12 months following discharge. After adjusting for clinical and demographic factors, we found that weekend admission was significantly associated with reduced length of admission and a slightly increased frequency of readmission. This may reflect the fact that patients admitted at the weekend have different clinical characteristics to those admitted during the week. The reasons for psychiatric hospital admission may relate to a lack of adequate support in the community, stressful life events, risk of harm to self or others and deteriorating mental state (which could be complicated by exposure to alcohol and illicit substances).^29–31^ A relatively brief weekend admission may provide support during an acute crisis but be less likely to address the underlying factors in the community that predispose the patient to further admissions in the future. Thus, the increased frequency of subsequent hospital admissions among those admitted during the weekend may reflect the fact that these patients (who are discharged earlier than those admitted during the week) had unresolved treatment needs. Although we found no difference in in-patient mortality between patients admitted at the weekend and during the week, patients who experience frequent readmissions may have an increased risk of worse clinical outcomes and mortality in the long-term.

### Strengths and limitations

The principal strength of our study is the large sample size of over 45 000 patients receiving psychiatric hospital care in a large geographic catchment area. By utilising an electronic case register covering all the psychiatric hospitals in SLaM, our study benefits from complete mortality and admission outcome data allowing analysis of these factors for all patients included in the study. Furthermore, through manual review of unstructured free-text clinical assessments in a subset of admissions we were also able to analyse data on seclusion and violent incidents, both of which are important adverse outcomes that may be associated with psychiatric hospital admission.

Balanced with these strengths are the limitations of using routine electronic health record data to analyse clinical outcomes. In our observational study we were unable to characterise reasons for hospital admission at an individual patient level or to obtain comprehensive data on predisposing and precipitating factors such as lack of support in the community, exposure to alcohol and illicit substances, diagnosis, illness severity and change in mental state leading up to admission.^29,30^ These factors may have influenced both the decision to admit to hospital as well as subsequent clinical outcomes that could have contributed to residual confounding in our study. As a consequence we were only able to analyse a small number of covariates in multivariable regression analyses. Future studies obtaining data on a wider range of potentially confounding variables included in a propensity score analysis may help to better explain the associations of weekend psychiatric hospital admission with clinical outcomes.

A further limitation is the lack of data on healthcare service factors such as ward staffing levels and bed availability.^32,33^ We were also unable to obtain data on the availability of community mental health services and social services. Variability in these resources on different days of the week are likely to have influenced likelihood of weekend admission and any subsequent clinical outcomes. Further studies including data on the availability of community mental health and social services on different days of the week would help to establish whether this contributes to variation in weekend clinical outcomes.

We found that patients were more likely to have been admitted from an acute hospital at the weekend than during the week. This could indicate an increased likelihood of presenting to an emergency department with an acute crisis at the weekend compared with during the week. Alternatively, this finding could reflect a lack of alternative services to provide community mental healthcare to people who present to emergency departments with an acute crisis at the weekend. We were unable to obtain data on the number of people presenting to emergency departments with an acute mental health crisis and the proportion of patients who were subsequently admitted to a psychiatric hospital. An analysis of people presenting to emergency department on different days of the week and the proportion of these who are subsequently admitted to a psychiatric hospital would help to determine the likely explanation for this finding. Although we analysed a large number of psychiatric hospital admissions, the extent to which our findings are generalisable to other mental healthcare providers remains uncertain. The risk of developing a mental disorder leading to hospital admission depends on a number of sociodemographic factors that vary in different healthcare settings.

### Implications

Our findings suggest that patients admitted to a psychiatric hospital at the weekend are not at greater risk of in-patient mortality compared with patients admitted during the working week. However, they are more likely to have brief admissions and to subsequently be readmitted suggesting that patients admitted at the weekend are more likely to have ongoing unresolved treatment needs. This difference may be partly related to demographic and clinical differences between patients admitted at the weekend compared with during the week. However, the findings may also reflect lack of availability of primary care, community mental health and social care services at the weekend. A recent report from the Independent Mental Health Taskforce to the NHS in England outlines an aspiration for good-quality mental healthcare on all 7 days of the week.^34^ Our study highlights the need to consider not only psychiatric hospital care, but also access to primary care, community mental health and social care services when planning service provision across the 7-day week.
